# GB Virus B and Hepatitis C Virus, Distantly Related Hepaciviruses, Share an Entry Factor, Claudin-1

**DOI:** 10.1128/jvi.00469-23

**Published:** 2023-06-13

**Authors:** Kamilla Toon, Mphatso D. Kalemera, Machaela Palor, Nicola J. Rose, Yasuhiro Takeuchi, Joe Grove, Giada Mattiuzzo

**Affiliations:** a Science Research and Innovation, Medicines and Healthcare Products Regulatory Agency, South Mimms, United Kingdom; b Division of Infection and Immunity, University College London, London, United Kingdom; c MRC-University of Glasgow Centre for Virus Research, Glasgow, United Kingdom; University of Southern California

**Keywords:** claudin-1, GB virus B, hepatitis C virus, virus entry

## Abstract

Due to increased and broadened screening efforts, the last decade has seen a rapid expansion in the number of viral species classified into the *Hepacivirus* genus. Conserved genetic features of hepaciviruses suggest that they have undergone specific adaptation and have evolved to hijack similar host proteins for efficient propagation in the liver. Here, we developed pseudotyped viruses to elucidate the entry factors of GB virus B (GBV-B), the first hepacivirus described in an animal after hepatitis C virus (HCV). GBV-B-pseudotyped viral particles (GBVBpp) were shown to be uniquely sensitive to the sera of tamarins infected with GBV-B, validating their usefulness as a surrogate for GBV-B entry studies. We screened GBVBpp infection of human hepatoma cell lines that were CRISPR/Cas9 engineered to ablate the expression of individual HCV receptors/entry factors and found that claudin-1 is essential for GBV-B infection, indicating the GBV-B and HCV share an entry factor. Our data suggest that claudin-1 facilitates HCV and GBV-B entry through distinct mechanisms since the former requires the first extracellular loop and the latter is reliant on a C-terminal region containing the second extracellular loop. The observation that claudin-1 is an entry factor shared between these two hepaciviruses suggests that the tight junction protein is of fundamental mechanistic importance during cell entry.

**IMPORTANCE** Hepatitis C virus (HCV) is a major public health burden; approximately 58 million individuals have chronic HCV infection and are at risk of developing cirrhosis and liver cancer. To achieve the World Health Organization’s target of eliminating hepatitis by 2030, new therapeutics and vaccines are needed. Understanding how HCV enters cells can inform the design of new vaccines and treatments targeting the first stage of infection. However, the HCV cell entry mechanism is complex and has been sparsely described. Studying the entry of related hepaciviruses will increase the knowledge of the molecular mechanisms of the first stages of HCV infection, such as membrane fusion, and inform structure-guided HCV vaccine design; in this work, we have identified a protein, claudin-1, that facilitates the entry of an HCV-related hepacivirus but with a mechanism not described for HCV. Similar work on other hepaciviruses may unveil a commonality of entry factors and, possibly, new mechanisms.

## INTRODUCTION

Hepaciviruses are classified into the *Flaviviride* family, which is a broad family of enveloped positive-strand RNA viruses. The *Hepacivirus* genus includes hepatitis C virus (HCV), a significant human pathogen causing an estimated 1.5 million new infections each year, with 58 million people being chronically infected globally (1, 2). GB virus B (GBV-B) was the second hepacivirus described and long remained the only HCV homolog known until another animal hepacivirus was isolated from the nasal swab of a dog with a respiratory illness in 2011 (3). Since then, explorations of potential animal hosts have uncovered hepaciviral sequences in a diverse range of hosts, including dogs, horses, bats, monkeys, rodents, cows, ticks, and sharks (4–10). Studies on these related viruses, including GBV-B, have highlighted the broad spread of hepaciviruses in the animal kingdom and have offered some clues into the origins of HCV (11). Indeed, the characterization of the life cycle of these viruses and the identification of other hepaciviruses could be the key to understanding their cross-species transmission and zoonotic potential. Furthermore, animal models of hepaciviral disease could be important surrogate models for HCV disease pathology and for screening the efficacy of vaccine candidates (12).

GBV-B was isolated from tamarin monkeys that had been experimentally inoculated with the serum of a surgeon (with the initials G. B.) who had presented with symptoms of acute hepatitis (13–15). However, the natural host of GBV-B remains enigmatic because of the lack of compelling evidence that humans and chimpanzees are susceptible to infection, indicating that the virus most likely did not come from the surgeon. In fact, GBV-B has yet to be isolated from any animals other than those infected experimentally (14, 16). Nonetheless, due to HCV’s restricted host tropism, infection of small New World monkeys (tamarins, marmosets, and owl monkeys) with GBV-B has previously been employed as a surrogate to study HCV disease and correlates of protection (17–21). GBV-B, like HCV, is associated with hepatitis and is found primarily in the liver of the infected host (22, 23). However, in contrast to HCV, GBV-B does not appear to cause chronic disease as the infection is usually cleared within 6 months (16, 23–25).

The lack of viral persistence, the high costs, and the implementation of the “three R’s” principle in animal research have lessened GBV-B infection in New World monkeys as a model for HCV; nevertheless, this model had, and still has, a role in the discovery and evaluation of antivirals and therapeutics for HCV. The tamarin model was key for determining the functional importance of genomic features such as the microRNA-122 binding site (26) and for screening for HCV antivirals such as ribavirin and NS3 protease inhibitors (27, 28). Furthermore, HCV/GBV-B chimeras that were developed to overcome the poor sequence identity between the two viruses may yet prove useful in B-cell vaccine candidate screens. These chimeras contain HCV-derived E1E2 glycoproteins (the main targets of anti-HCV antibodies), can infect marmosets chronically, and show liver pathology consistent with that of HCV in humans (18). Therefore, marmoset disease progression could be monitored to assess the efficacy of candidate vaccines.

The hepatotropic nature of most mammalian hepaciviruses described to date suggests that these viruses (or common ancestors) have undergone a high degree of evolutionary adaptation to the liver. For instance, the 5′ untranslated regions of most mammalian hepacivirus sequences contain putative binding sites for microRNA-122, which is liver specific (11). It can be inferred that hepaciviruses may exploit various orthologous host factors for efficient propagation in the liver. Therefore, HCV’s specific molecular interactions with host factors may be conserved in other hepaciviruses.

A virus, being an obligatory parasite, needs to enter the host cell. HCV exhibits a complex entry mechanism, involving at least four host factors, which is not fully understood. The virion attaches to hepatocytes via heparan sulfate proteoglycans; at this point, E1E2 glycoproteins engage scavenger receptor class B type 1 (SR-B1) and the cluster of differentiation 81 (CD81) molecule (29, 30). CD81 engagement is thought to initiate a signaling cascade that leads to intracellular actin remodeling, driving the translocation of the CD81-tethered virus toward the tight junction. HCV then acquires claudin-1 (CLDN1) and occludin (OCLN) during transit to or at the tight junction (31, 32). Finally, the virion is internalized via clathrin-mediated endocytosis, and the process culminates when the low-pH environment of the early endosome promotes E1E2-catalyzed fusion between the viral and endosomal lipid bilayers (33). Other entry factors such as low-density lipoprotein (LDL) receptor (LDLR), epidermal growth factor receptor (EGFR), ephrin receptor A2 (EphA2), and Niemann-Pick C1-like 1 (NPC1L1) have been identified as cofactors for entry (33–36).

Recently, SR-B1 has been shown to mediate the entry of a cell culture-derived rat hepacivirus (37). Aside from this and the HCV entry factors outlined above, no other entry factors have been identified for other hepaciviruses. *In vitro* studies have been impaired by the scarcity of replication-competent full-length cell culture viruses. However, the discovery of novel hepaciviral entry factors could reveal mechanistic details surrounding HCV entry and potentially inform vaccine design. Moreover, such studies could uncover a novel mechanism of lipid bilayer fusion as hepaciviral E1E2 glycoproteins are genetically and structurally predicted to belong to a novel class of membrane fusion proteins, outside the three described so far (38). In this report, we adapted the well-established HCV-pseudotyped viral particles (HCVpp) system to study GBV-B cell entry. HCVpp are retrovirus-based particles bearing HCV E1E2 glycoproteins. HCVpp were crucial in the identification of CLDN1 and OCLN as HCV entry factors (39–41) and the determination of epitopes that are targeted by neutralizing antibodies during infection (42–44). Here, we generated GBV-B-pseudotyped viral particles (GBVBpp) and then employed a receptor knockout (KO) cell line screen to characterize GBV-B entry. We found that, like HCV, GBV-B entry is also dependent on CLDN1 but that the two viruses rely on different CLDN1 domains to enter the cell, suggesting subtle mechanistic differences.

## RESULTS

### Production of GBV-B-pseudotyped virus.

**FIG 1:**
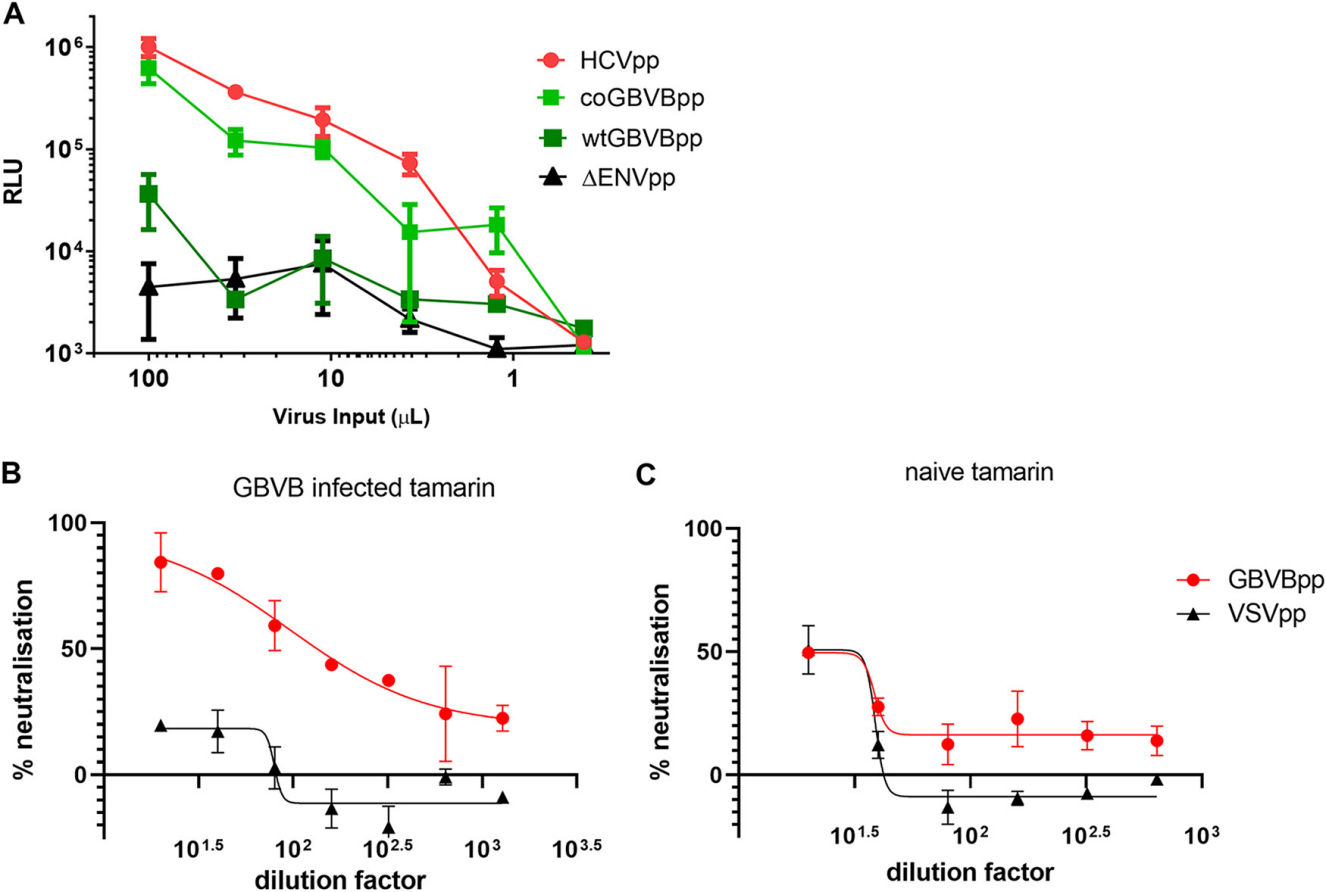
Production of MLV core pseudotyped with GBV-B E1E2. (A) HCVpp (isolate UKN1A20.8); GBVBpp, which were produced using a plasmid with GBV-B E1E2 sequences cloned from an ATCC stock (wtGBVBpp) or codon optimized for mammalian expression (coGBVBpp); and ΔENVpp were produced with an MLV vector containing a luciferase reporter gene. Producer cell supernatants containing particles were titrated on Huh 7.5 cells. (B and C) GBVBpp, with the codon-optimized E1E2 sequence, were incubated with serial dilutions of serum from a tamarin experimentally infected with GBV-B (B) or a naive animal (C) prior to infection of Huh7.5 cells. An MLV vector pseudotyped with vesicular stomatitis virus glycoprotein G (VSVpp) was also tested against the same tamarin sera to confirm the specificity of neutralization. The percentages of neutralization reported are the means from 2 independent experiments in triplicate, fitted onto a 4-parameter logistic curve model.

To study and compare the entry mechanisms of GBV-B and HCV, a retroviral vector based on murine leukemia virus (MLV), containing a firefly luciferase reporter gene, was produced, similar to previous work (21). The infectivity of the HCV-pseudotyped viral particles (HCVpp) and the GBV-B-pseudotyped viral particles (GBVBpp) was assessed on an immortalized human cell line, Huh7.5, and detected as a luminescence signal (Fig. 1A). Infectivity over the background at a level similar to that of HCVpp could be obtained only when GBVBpp were produced using codon-optimized E1E2 sequences, which were therefore chosen for the production of GBVBpp. These pseudotyped particles were then tested against archived serum from a tamarin experimentally infected with GBV-B (17) (Fig. 1B). The infectivity of GBVBpp was inhibited in a dose-dependent manner by sera from GVB-B-infected tamarins but not by serum from a naive tamarin (Fig. 1C); this is consistent with specific entry being driven by GBV-B E1E2 and indicates that GBVBpp represent a suitable model for entry and serological studies.

### CLDN1 mediates GBV-B entry into human cells.

**FIG 2:**
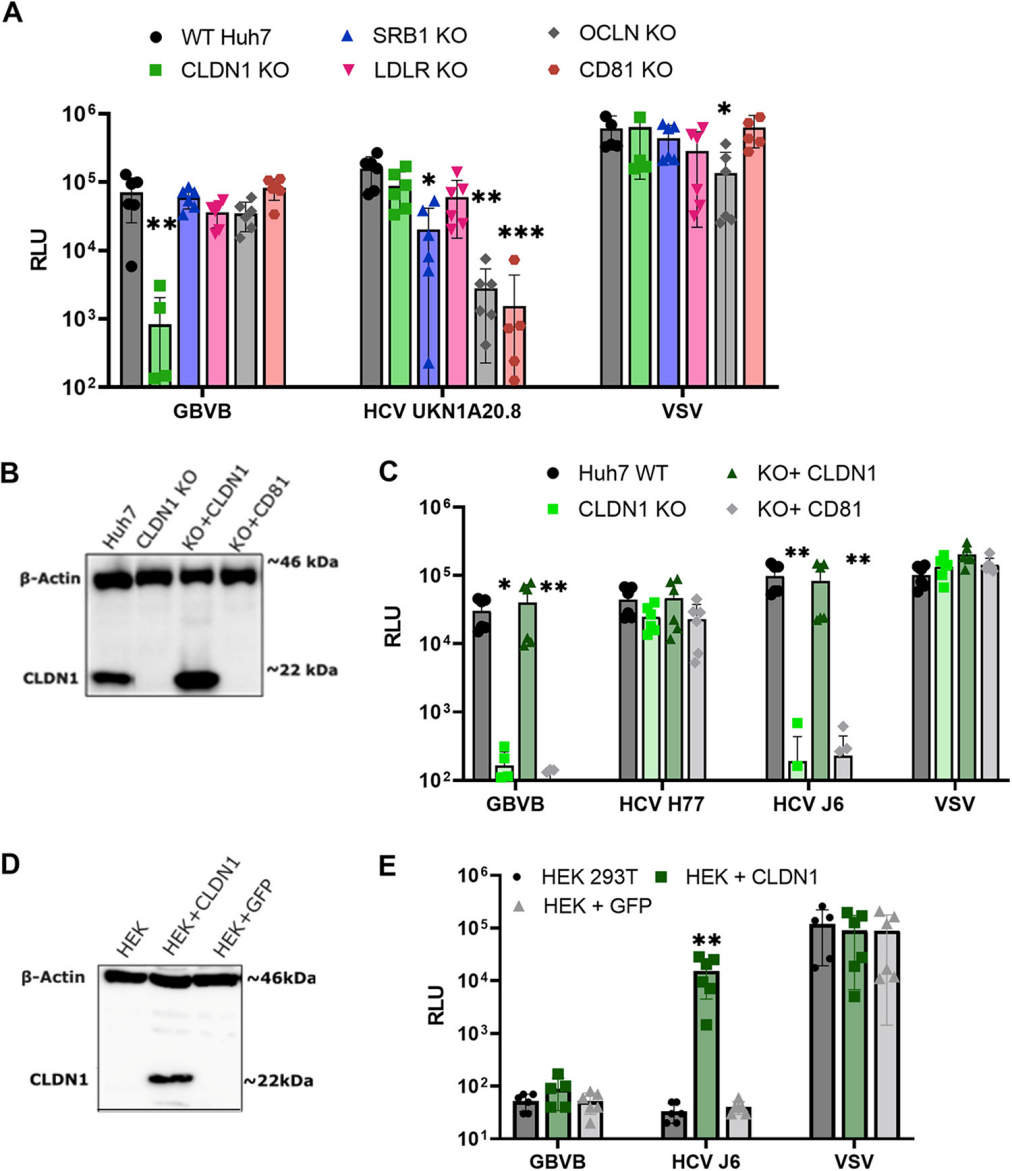
CLDN1 is required for GBV-B entry into human cells. (A) Huh7 cells in which CLDN1, SR-B1, LDLR, OCLN, or CD81 was knocked out were infected with GBVBpp, HCVpp, or VSVpp. (B and D) CLDN1 expression in modified Huh7 (B) or HEK293T (HEK) (D) cells was assessed by immunoblotting using an anti-CLDN1 antibody. The protein input was verified using an anti-actin antibody. Samples were run with a protein size marker; the size (in kilo daltons) is indicated at the right of the blot. The blots are consistent with the predicted molecular weight of CLDN1 being 23 kDa. (C) Huh7 cells and Huh7 CLDN1 KO cells transduced with lentiviral vectors to express CLDN1 or CD81 were challenged with the indicated pseudotyped viruses. (E) HEK293T cells were transduced to express CLDN1 and/or an irrelevant protein (GFP) and challenged with GBV-B-, HCV-, or VSV-pseudotyped virus. Infectivity is expressed as mean RLU values ± standard deviations from 2 independent experiments run in triplicate. *, *P* < 0.05; **, *P* < 0.005; ***, *P* < 0.0005 (by a Kruskal-Wallis test compared to the wild type [WT]).

Given that GBV-B is closely related to HCV and is also hepatotropic, we hypothesized that the two viruses may share conserved entry factors. To this end, a panel of HCV receptor knockout (KO) Huh7 cell lines (45) was screened for susceptibility to GBVBpp entry (Fig. 2A). No effect was observed using CD81, OCLN, LDLR, or SR-B1 KO cell lines; however, GBVBpp entry was significantly reduced in CLDN1 KO cells (Fig. 2A). CLDN1 KO did not significantly impact HCVpp entry; this is because the E1E2 glycoproteins used for pseudotyping in this experiment were derived from an HCV isolate (UKN1A20.8) that is able to utilize CLDN6 and CLDN9 in addition to CLDN1 (36). HCVpp showed a significant decrease in cell entry in SR-B1, CD81, OCLN KO, and, to a lesser extent, LDLR KO, which was expected as LDLR is used redundantly with SR-B1. An unrelated pseudotyped MLV carrying vesicular stomatitis virus glycoprotein G (VSV-G) (VSVpp) was used as a negative control and infected CLDN1 KO cells as efficiently as it infected unmodified Huh7 cells.

To further confirm the role of CLDN1 in GBV-B entry, CLDN1 expression in KO cells was reconstituted to determine if this restored susceptibility to GBVBpp. CLDN1 KO cells were transduced with a lentiviral vector carrying CLDN1 or an irrelevant gene (CD81) (Fig. 2B). The exogenous expression of CLDN1, but not CD81, restored the susceptibility of KO cells to GBVBpp and HCVpp of J6, a CLDN1-restricted strain of HCV, to wild-type levels (Fig. 2C). This result further supports that CLDN1 is necessary for GBV-B entry. Also, the expression of CLDN1 above endogenous levels did not enhance entry, consistent with what was observed previously for HCV entry (31). HCVpp carrying isolate H77-derived E1E2 could similarly infect CLDN1 KO and wild-type cells, consistent with previous findings that this isolate can use CLDN6 or CLDN9 as well as CLDN1 for entry (46).

HEK293T cells are nonpermissive to HCV entry as they do not express CLDN1. However, upon the exogenous expression of CLDN1, HEK293T cells become highly permissive to HCVpp, indicating that all other HCV entry factors are present (30). CLDN1-transduced HEK293T cells (Fig. 2D) were susceptible to HCVpp infection, whereas no signal was detected for GBVBpp (Fig. 2E). This observation suggests that there is at least one other entry factor present in Huh7, but not HEK293T, cells that is essential for GBV-B entry, consistent with its liver tropism. Alternatively, there may be unknown inhibitory factors in HEK293T cells specific to GBV-B but not HCV.

### CLDN1 regions critical for GBV-B entry differ from those required for HCV entry.

**FIG 3:**
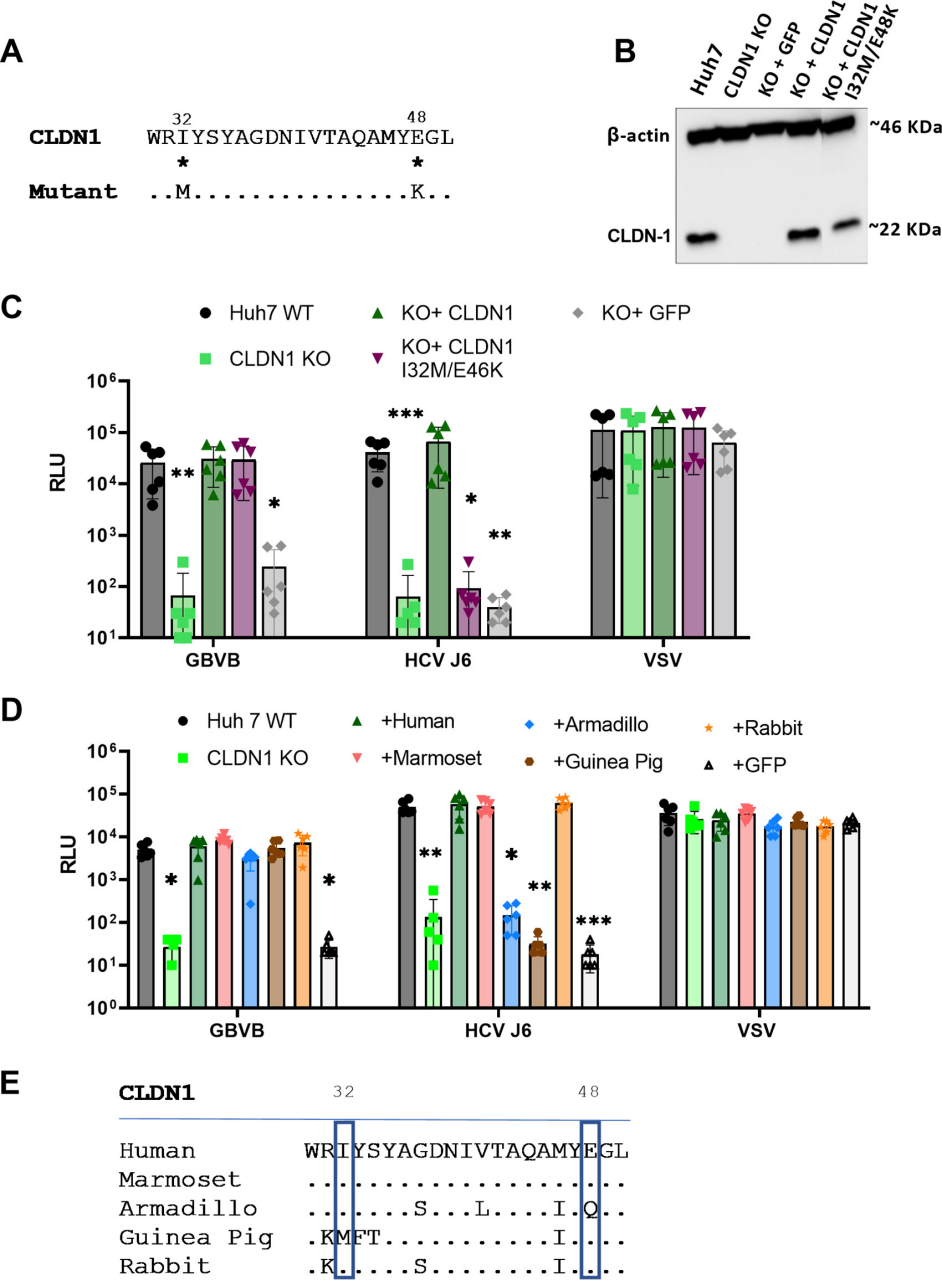
Different CLDN1 regions are important for GBV-B and HCV entry. (A) Alignment of CLDN1 amino acids 30 to 50 in extracellular loop 1 with the mutant created by site-directed mutagenesis to introduce the I32M and E48K mutations. Identical amino acids are represented by a full stop, and the numbering represents the amino acid position in full-length CLDN1 (GenBank accession no. NP_066924.1). (B) CLDN1 expression in modified Huh7 cells was assessed by immunoblotting using an anti-CLDN1 antibody. The protein input was verified using an anti-actin antibody. Samples were run with a protein size marker; the size (in kilo daltons) is indicated at the right of the blot. (C and D) Huh7 CLDN1 KO cells were transduced to express CLDN1, CLDN1 I32M/E48K, or GFP only (C) or the indicated mammalian CLDN1 (D) and challenged with HCV J6pp, GBVBpp, or VSVpp. Infectivity is expressed as mean RLU values ± standard deviations from 2 independent experiments run in triplicate. *, *P* < 0.05; **, *P* < 0.005; ***, *P* < 0.0005 (by a Kruskal-Wallis test compared to the wild type [WT]). (E) Alignment of human CLDN1 amino acids 30 to 50 in extracellular loop 1 with the same regions of the selected mammalian CLDN1 proteins. Identical amino acids are represented by a full stop, and the numbering represents the amino acid position in full-length human CLDN1.

Claudin-1 is a small protein (211 amino acids [aa]) located at the cell surface with 4 transmembrane domains and 2 extracellular loops, with both the N and C termini being intracellular (47). Previous work demonstrated that amino acid residues at positions 32 and 48 in the first extracellular loop (EL1) of CLDN1 are crucial for supporting HCV entry (31). We sought to determine whether the same residues are similarly important for GBV-B infection. Through site-directed mutagenesis, we generated a lentiviral vector for a CLDN1 mutant expressing an isoleucine-to-methionine substitution at position 32 (I32M) and a glutamic acid-to-lysine substitution at position 48 (E48K) in CLDN1 EL1 (Fig. 3A). The I32M/E48K mutant was then introduced into CLDN1 KO cells by lentiviral transduction and confirmed by Western blotting (Fig. 3B). The mutant, as expected, failed to rescue HCV J6pp entry (Fig. 3C). Notably, mutant and wild-type CLDN1 proteins restored GBVBpp infectivity to similar degrees (Fig. 3C), indicating that I32 and E48 of CLDN1 are not essential for GBV-B’s cell entry. Further confirmation was obtained by looking at CLDN1 proteins from other species. CLDN1 KO cells were transduced to express mammalian CLDN1; armadillo and guinea pig CLDN1 did not support HCV J6pp entry, while rabbit and marmoset CLDN1 allowed infection (Fig. 3D). This is likely due, at least in part, to the residue changes in amino acid I32 in guinea pig CLDN1 and amino acid E48 in armadillo CLDN1 (Fig. 3E). As shown for the human CLDN1 mutant, all of the tested mammalian CLDN1s conferred susceptibility to GBV-B (Fig. 3D). This result also suggests that CLDN1 on its own is not a determinant of host range. It has been reported that CLDN1 is also not a species specificity determinant for HCV, as murine CLDN1 confers susceptibility to HCV in HEK293T cells (31).

### Characterization of CLDN1 regions important for GBV-B entry.

**FIG 4:**
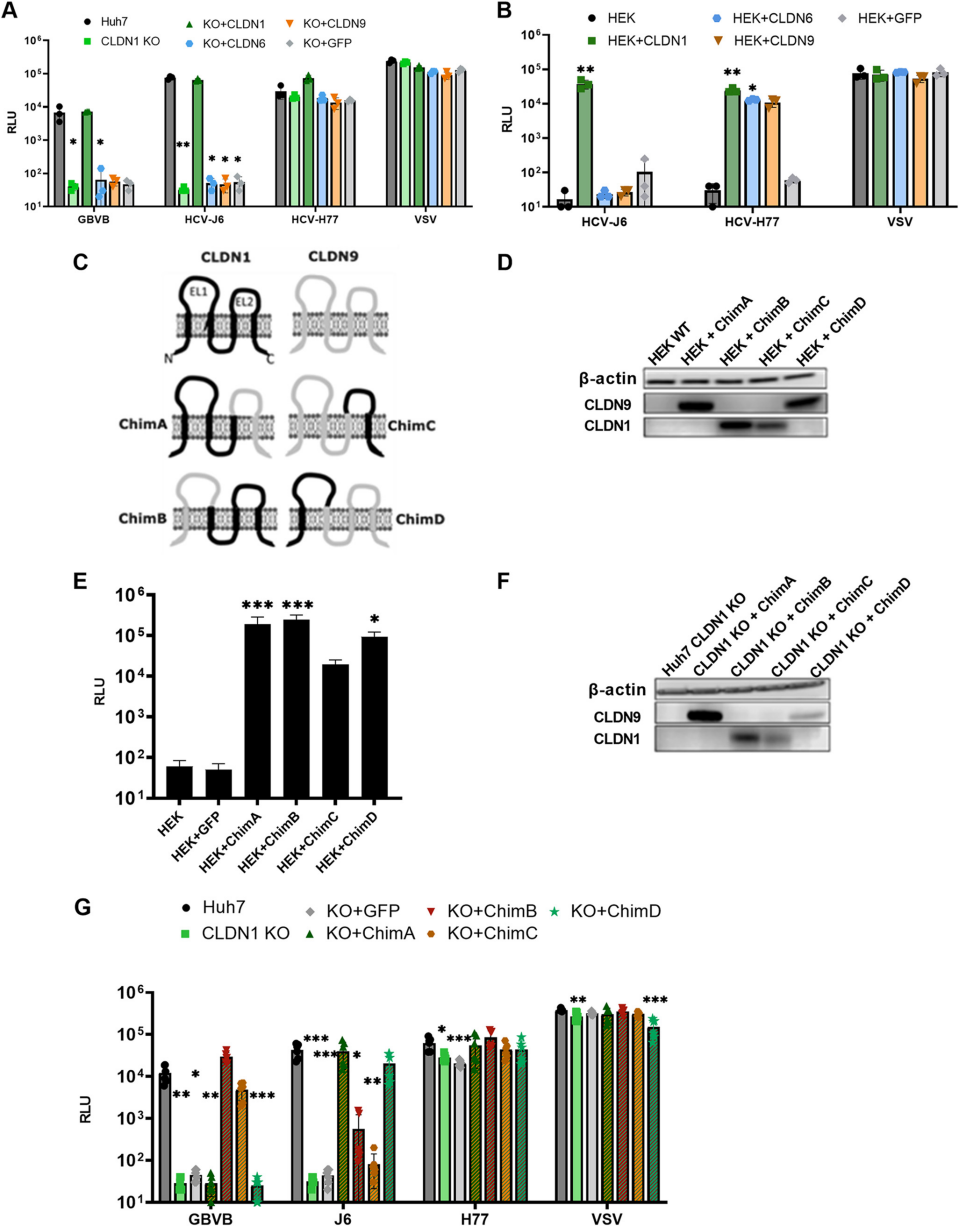
Extracellular loop 2 of claudin-1 is important for GBV-B entry. (A and B) Huh7 CLDN1 KO (A) or HEK293T (B) cells transduced to express CLDN1, CLDN9, CLDN6, or GFP were challenged with pseudotyped viruses harboring glycoproteins from GBV-B, HCV J6, HCV H77, and VSV. (C) Claudin-1 (black) and claudin-9 (gray) topologies with the indicated regions of each protein swapped with the respective regions of the other CLDNs to produce chimeric proteins, resulting in chimeric proteins with the following amino acids: (CLDN1 aa 1 to 139/CLDN9 aa 138 to 217) (ChimA), CLDN9 aa 1 to 81/CLDN1 aa 82 to 211 (ChimB), CLDN9 aa 1 to 137/CLDN1 aa 139 to 211 (ChimC), and CLDN1 aa 1 to 81/CLDN9 aa 82 to 217 (ChimD). (D and F) Chimeric protein expression in HEK293T cells (D) or Huh7 cells (F) was assessed by immunoblotting using anti-CLDN1 and anti-CLDN9 antibodies. The protein input was verified using an anti-actin antibody. (E) HEK293T cells transduced to express the indicated chimeric CLDN1/9 proteins were infected with H77pp. (G) Huh7 CLDN1 KO cells transduced to express the indicated chimeric CLDN1/9 proteins were infected with GBVBpp, J6pp, J77pp, and VSVpp. Infectivity is expressed as the mean RLU values ± standard deviations from 2 independent experiments run in triplicate. *, *P* < 0.05; **, *P* < 0.005; ***, *P* < 0.0005 (by a Kruskal-Wallis test compared to the wild type [WT]).

We next sought to determine which region of CLDN1 is important for GBV-B entry using domain-swapping studies. CLDN6 and CLDN9 were selected as nonpermissive candidates; this choice was driven by the observation that CLDN1-independent HCV strain H77 can enter CLDN1 KO cells (Fig. 2B), which indicates that CLDN6 and/or CLDN9 is expressed in Huh7 cells but that GBVBpp are unable to utilize it for cell entry. The CLDN6 and CLDN9 genes were subcloned from Huh7 cells into a lentiviral vector and used to transduce CLDN1 KO cells. Only the addition of CLDN1, and not the overexpression of CLDN6 or CLDN9, conferred susceptibility to GBVBpp and HCV J6pp in KO cells (Fig. 4A). HCV H77pp infection of CLDN6- and CLDN9-transduced HEK293T cells confirmed that the ectopic protein was correctly folded and functional (Fig. 4B). CLDN9 was chosen for domain-swapping experiments as it has slightly higher homology to CLDN1 at the amino acid level than CLDN6, 45% versus 43%. An overlapping PCR strategy was employed to generate CLDN1/CLDN9 chimeras reciprocally swapped for their extracellular loops (EL1 and EL2) (Fig. 4C).

The chimeric CLDN1/9 proteins were initially transduced into HEK293T cells, and their expression was confirmed by Western blotting (Fig. 4D). The anti-CLDN1 and anti-CLDN9 antibodies appear to recognize a region in the C termini of their respective proteins; therefore, chimera A (ChimA) and ChimD can be detected by anti-CLDN9 antibodies, and ChimC and ChimB can be detected by anti-CLDN1 antibodies. To assess the functionality of these chimeric CLDN1/9 proteins, transduced HEK293T cells were challenged with HCV H77pp, which use both CLDN1 and CLDN9. Figure 4E shows the complete panel of chimeric CLDN1/9 proteins expressed in HEK293T cells, which conferred susceptibility to H77pp, while the parental cells and the transduction control (expressing green fluorescent protein [GFP] only) remained uninfectable by the virus. This confirms that all chimeras were conformationally correct and supported HCV infection.

The panel of chimeras was then expressed in Huh7 CLDN1 KO cells (Fig. 4F) and tested for susceptibility to GBVBpp and J6pp (Fig. 4G). We found that GBVBpp could infect CLDN1 with EL1 replaced with that of CLDN9 (ChimB), but infection fell below the limit of detection when EL2 was swapped (ChimA), suggesting that the C-terminal region containing EL2 of CLDN1 is critical for GBV-B entry. Consistently, GBVBpp infection was observed when EL2 of CLDN1 was introduced into CLDN9 (ChimC). Furthermore, no infectivity was observed when EL1 was introduced (ChimD), confirming that EL2, or a region downstream, is the critical region. The opposite was observed for HCV J6pp, as expected, as HCV is known to utilize EL1 (31). The difference in the CLDN1 regions necessary for GBV-B and HCV infection suggests a novel mechanism of interaction with CLDN1 compared to that described for HCV.

## DISCUSSION

Much of what is known of the hepaciviral life cycle is inferred from HCV, which is by far the most extensively studied virus of the genus due to the significant disease burden that it poses to humans. GBV-B was sequenced in 1995 and since then has been a useful model for HCV research (18, 26–28); despite this, no receptors or entry factors had been described for it. Here, we demonstrate that like HCV, CLDN1 is an entry factor that is necessary for GBV-B cell entry. When CLDN1 is knocked out of the Huh7 line, a cell line susceptible to GBVBpp, entry is diminished (Fig. 2A), and upon the exogenous expression of CLDN1, susceptibility is completely restored (Fig. 2B). Huh7 cells with CD81, OCLN, LDLR, or SR-B1 knocked out had no impact on GBVBpp entry.

In addition to HCV, coxsackievirus B, some reoviruses, and some adenoviruses also utilize tight junction proteins as entry cofactors (48, 49). In polarized cells, the majority of CLDN1 is localized in tight junctions (31). HCV is thought to follow a cell entry pathway similar to that of coxsackievirus B, where it binds a primary receptor on the luminal cell surface and then migrates laterally along the plasma membrane to encounter the tight junction proteins CLDN1 and OCLN (33). However, there is a small amount of CLDN1 that localizes to the basal membranes of hepatocytes, and there is evidence to support that HCV can utilize this fraction of CLDN1 (50–52). Of note, GBVBpp were insensitive to OCLN deletion, which may further highlight a divergence in the entry pathways of HCV and GBV-B since OCLN, which localizes exclusively to the tight junctions, is indispensable for HCV infection.

Two amino acids in extracellular loop 1 (EL1) of CLDN1, previously identified as being essential for HCV entry (31), have no impact on GBVBpp infection (Fig. 3B). These residues are responsible for the interaction of CLDN1 with CD81 to form a complex that is indispensable for HCV entry (53). It is not surprising that these residues are not important for GBV-B entry because CD81 does not appear to play a role in GBV-B entry into human cells (Fig. 2A); thus, the inability to form this complex was expected to have no effect, further confirming that GBV-B entry is independent of CD81. Marnata and colleagues have shown that HCV entry into tamarin cells is dependent on tamarin CD81 (21). If GBV-B cell entry was dependent on CD81 in a manner similar to that of HCV, then a reduction in GBVBpp infectivity in CD81 KO cells would be expected, which was not observed in this study. We have also shown that GBV-B entry is dependent on EL2 of CLDN1 or a region downstream. Using chimeric proteins between permissive CLDN1 and nonpermissive CLDN9, GBVBpp were able to enter only those cells expressing a chimeric CLDN containing CLDN1 EL2 and the downstream region, while CLDN1-dependent HCV strain J6pp were able to infect cells expressing chimeras with EL1 of CLDN1 (Fig. 4G). Further investigation is needed to determine the specific residues of CLDN1 that are important for GBV-B entry and whether GBV-B interacts directly with CLDN1.

It was initially thought that CLDN1 does not interact directly with HCV particles but is important for the receptor complex formed with CD81 (53, 54). However, some studies have indicated that there may be a direct interaction between CLDN1 and the HCV E1E2 complex. CLDN1 has been shown to interact not with E2 alone but with the E1E2 heterodimer (55), and mutations in E1 have been shown to shift the use of CLDN1 to CLDN6 (56, 57), indicating a direct interaction with E1. However, this interaction is poorly understood; it is not known which domains of CLDN1 are important for the interaction with the glycoproteins or if binding to CLDN1 is needed for receptor clustering. It is also currently unknown whether CLDN1 binds to E1 or whether the E1E2 glycoproteins together form a conformational domain for interaction.

Another discrepancy observed between HCVpp and GBVBpp is the ability to infect HEK293T cells transduced to express CLDN1: they are susceptible to HCVpp but not GBVBpp (Fig. 3E). This suggests that GBV-B may utilize at least one entry factor not conserved between GBV-B and HCV that is expressed in Huh7 cells but not HEK293T cells. Thus, further investigation into other entry factors is needed to fully characterize GBV-B cell entry, for example, through a CRISPR screen of liver-enriched cell membrane proteins. The identification of further receptors or entry factors that are not conserved may shed light on the physiological differences seen between the New World monkey animal models for GBV-B and HCV infection. Alternatively, HEK293T, but not Huh7, cells may have a factor restricting GBV-B entry.

A caveat to our investigations is that GBVBpp may not be representative of authentic replicating viral particles. Being closely related to and displaying a similar hepatotropism, it is likely that GBV-B particles, like HCV, resemble low-density lipoprotein complexes associated with apolipoproteins (58, 59); therefore, apolipoprotein receptors such as LDLR and SR-B1 could play a significant role in virus entry as they can tether apolipoprotein-associated virions to the basolateral surface of hepatocytes (30, 34, 45). Indeed, it was recently shown by Wolfisberg and colleagues that an infectious cell culture-derived rat hepacivirus also shared biophysical properties with LDL and was dependent on SR-B1 for entry (37). To date, it has not been reported that GBV-B replicates in any immortalized cell line tested, including Huh7 cells (60). The development of culture-derived replication-competent GBV-B *in vitro* and subsequent ultrastructural analyses of this virus could help establish whether the cooption of the LDL biogenesis pathway is a feature that is conserved among hepaciviruses. Additionally, the findings of this study would be strengthened if they were confirmed in hepatic cell lines derived from New World monkeys susceptible to GBV-B infection. This would, however, require the development of receptor knockout monkey cell lines or antibodies to the cognate monkey receptors, which goes beyond the scope of the current study.

GBV-B provides a potential avenue to investigate the interaction between E1E2 glycoproteins and CLDN1 without reliance on the CD81 complex, which could help clarify CLDN1’s role in HCV cell entry. Characterization of the entry mechanism may unveil new potential targets for therapeutics and uncover a novel mechanism of membrane fusion. Unlike flavivirus entry protein E, hepaciviral E1E2 proteins are not class II membrane fusion proteins; indeed, structural analyses suggest that hepacivirus E1E2 may represent a novel class of fusion proteins (61–64). To this end, studying GBV-B E1E2 entry may prove to be the more facile path to deciphering the hepaciviral membrane fusion mechanism as it is structurally predicted to have fewer disordered segments than HCV E1E2 (65). Determining the hepaciviral fusion mechanism could inform structure-guided HCV vaccine design. Understanding the similarities and differences between HCV E1E2 and GBV-B E1E2 will likely provide mechanistic insight into HCV’s complex entry process. It may also offer clues as to why it seems that, among hepaciviruses, HCV has uniquely evolved to cause chronic disease in its host, while close relatives succumb to their hosts’ immune systems in the acute stages of infection.

In summary, we have discovered that two distantly related hepaciviruses, HCV and GBV-B, share CLDN1 as a cell entry factor, but their modes of CLDN1 usage are different. This suggests that the dependence on this entry factor may have arisen by convergence through two different evolutionary routes. Alternatively, the use of CLDN1 as an entry factor by a hepacivirus ancestor along with the establishment of liver tropism may have been retained with certain differentiation of the interaction mode by divergent extant hepaciviruses. We expect that future studies on cell entry by other animal hepaciviruses will shed some light on the evolutionary relationships between hepaciviruses and cellular entry factors.

## MATERIALS AND METHODS

### Cells.

HEK293T cells were obtained from the American Type Culture Collection (ATCC) (ATCC CRL-11268). Huh7 and HCV receptor knockout cell lines were kindly supplied by Yoshiharu Matsuura (Osaka University) (45). To genetically modify cell lines, 1 million Huh7 CLDN1 KO or HEK293T cells were seeded into 6-well plates, and approximately 2 h later, they were transduced with VSV-G-pseudotyped lentiviral vectors expressing GFP and the protein of interest at a multiplicity of infection (MOI) of approximately 1, in the presence of 8 μg/mL of Polybrene. All cells were maintained at 37°C with 5% CO_2_ in Dulbecco’s modified essential medium (DMEM) supplemented with GlutaMAX (Gibco), 10% fetal bovine serum (Pan Biotech), 100 U/mL penicillin, 100 μg/mL streptomycin (Sigma), and 1% nonessential amino acids (Gibco).

### Tamarin sera.

Archived sera from GBV-B-infected red-bellied tamarins (Saguinus labiatus) were available from a previous study (17).

### Production of pseudotyped viruses.

To produce lentiviral or gammaretroviral pseudotyped viruses, 4 million HEK293T cells were seeded into a 10-cm dish to reach 50 to 70% confluence. The next day, a DNA-plasmid mix was prepared, containing 1 μg of a plasmid encoding either MLV or HIV structural and enzymatic proteins, pCMVi (66) or p8.91 (67), respectively; 1 μg of a viral envelope protein expression plasmid; and 1.5 μg of a transfer vector plasmid expressing the luciferase gene (pCFCR-LUC) or the GFP reporter gene (pDual) in 15 μL of Tris-EDTA buffer. The DNA-plasmid mix was added to 18 μL of Fugene-6 transfection reagent (Promega) in 200 μL of prewarmed Opti-MEM (Gibco) and incubated for 20 min. The transfection mix was then added to the cells, which were incubated at 37°C with 5% CO_2_ for 24 h before the medium was replaced. After 48 h, the supernatant was collected and filtered through a 0.45-μm cellulose acetate membrane (BioWhittaker). The pseudotyped viral particles contained in the supernatant were concentrated at either 1,240 relative centrifugal force (RCF) overnight at 4°C or 103,586 RCF for 2 h at 4°C.

### Plasmids.

Full-length GBV-B E1E2, nucleotides 851 to 2449 (GenBank accession no. NC_001655.1), and codon-optimized E1E2 genes (GenBank accession no. OQ411604) were cloned into pCAGGS (68). pD607_J6_E1E2 (69) and pD603_H77_E1E2 (Addgene plasmid 86983) are mammalian expression vectors encoding the codon-optimized HCV glycoproteins from strains J6 and H77, respectively. pDual_CLDN1 or pDUAL_CD81 is a lentiviral dual-promoter transfer vector expressing GFP and CLDN1 or CD81, respectively (Addgene plasmids 86981 and 86980). Mutant (I32M and E48K) pDual_CLDN1 was created using a Q5 site-directed mutagenesis kit (New England BioLabs) with the following primers: I32M_F (5′-CCA GTG GAG GAT GTA CTC CTA TGC C-3′), I32M_R (5′-GGC AGG GCA GTG CTG ACG-3′), E48K_F (5′-GGC CAT GTA CAA GGG GCT GTG GA-3′), and E48K_R (5′-TGG GCG GTC ACG ATG TTG-3′). To clone the CLDN6 and CLDN9 genes, RNA from Huh7 cells was extracted using an RNeasy kit (Qiagen) according to the manufacturer’s instructions. cDNA was then synthesized from the RNA with a Superscript IV transcriptase kit (Invitrogen) according to the manufacturer’s instructions using the following primers: CLDN6_F (5′-AAT TAG GAT CCG CCG CCA CCA TGG CCT CTG CCG GAA TGC A-3′), CLDN6_R (5′-GCG GCG GCC GTC GAC TCA GAC GTA ATT CTT GGT AGG GTA-3′), CLDN9_F (5′-AAT TAG GAT CCG CCG CCA CCA TGG CTT CGA CCG GCT TAG A-3′), and CLDN9_R (5′-GCG GCG GTC GAC TCA CAC GTA GTC CCT CTT-3′). The amplified genes were subcloned into the pDual lentiviral vector using a BamHI restriction site at the 5′ end and a SalI restriction site at the 3′ end of the gene, introduced with the primers. Successful cloning was confirmed by Sanger sequencing.

### Chimeric protein production.

Chimeric proteins containing the sequences of the CLDN1 and CLDN9 proteins were spliced using PCR-driven overlap extension as described previously (70). Briefly, a first PCR was performed using the templates and primers shown in Table 1 with KOD hot start DNA polymerase (Sigma-Aldrich), with an annealing time of 30 s at the indicated temperatures. Primers A and B or primers C and D (Table 1) were designed to introduce an overlapping sequence of 10 to 12 nucleotides into each protein fragment that spans the junction where the proteins will be spliced together. In a second PCR, the overlapping sequences created by the first PCR were annealed to join the sequences of CLDN1 and CLDN9, and primers A and D were used to amplify the hybridized product. Correct product synthesis was verified by Sanger sequencing.

**TABLE 1:** Overlap extension PCR primers

Chimera	Template	Sequence (5′–3′)		Annealing temp (°C)
ChimA	CLDN1	Primer A: AAA AAG GAT CCG CCG CCA CCA TGG CCA ACG CGG GG	Primer B: TGC GCC GTC CAT GCT GTG GCA A	62
	CLDN9	Primer C: TTG CCA CAG CAT GGA CGG CGC A	Primer D: GCG GCG GTC GAC TCA CAC GTA GTC CCT CTT	62

ChimB	CLDN9	Primer A: AAT TAG GAT CCG CCG CCA CCA TGG CTT CGA CCG GCT TAG A	Primer B: ACC ATC AAG GCA CGT GCG GCC	64
	CLDN1	Primer C: AGG CCG CAC GTG CCT TGA TGG T	Primer D: GCC GTC GAC TCA CAC GTA GTC TTT CCC	60

ChimC	CLDN9	Primer A: AAT TAG GAT CCG CCG CCA CCA TGG CTT CGA CCG GCT TAG A	Primer B: TGC CAT ACC AGC ACA CAG GG	62
	CLDN1	Primer C: CCC TGT GTG CTG GTA TGG CA	Primer D: GCC GTC GAC TCA CAC GTA GTC TTT CCC	60

ChimD	CLDN1	Primer A: AAA AAG GAT CCG CCG CCA CCA TGG CCA ACG CGG GG	Primer B: GAC ACA GAG GGC ACG GGT TGC TTG	66
	CLDN9	Primer C: CAA GCA ACC CGT GCC CTC TGT GTC	Primer D: GCG GCG GTC GAC TCA CAC GTA GTC CCT CTT	66

### Immunoblotting.

Cells were resuspended in lysis buffer on ice for 5 min as previously described (71) and centrifuged for 5 min, and the supernatant was collected for analysis. Proteins were separated by SDS-PAGE in a 4 to 20% Tris-glycine gel. Proteins were transferred to a nitrocellulose membrane, and nonspecific binding was blocked by incubation with 2 % milk and 0.1 % Tween 20 in phosphate-buffered saline (PBS). Membranes were probed by serial incubation with rabbit anti-CLDN1 antibody (1:1,000; Abcam) or rabbit anti-CLDN9 antibody (1:300; Proteintech), rabbit anti-β-actin antibody (1:10,000; Abcam), and goat anti-rabbit secondary antibody conjugated to horseradish peroxidase (HRP). The chemiluminescence signal was then measured using the Chemidoc MP system (Bio-Rad) or a D-Digit blot scanner (Li-Cor).

### Infectivity assays.

For pseudotyped particles with a luciferase reporter, the target cells were seeded at 15,000 cells per well into a 96-well plate and infected with the supernatant containing pseudotyped particles, with a final concentration of 4 μg/mL of Polybrene. The plates were spin inoculated at 1,240 RCF for 30 min at 20°C. Approximately 72 h later, the cells were lysed by the addition of 100 μL of a 1:1 (vol/vol) mixture of phenol red-free DMEM and Bright-Glo substrate (Promega), and infectivity was determined as the relative luminescence units (RLU) per well using Glomax Navigator (Promega). For pseudotypes carrying a GFP reporter gene, target cells were seeded at 100,000 cells per well into a 24-well plate and transduced with the supernatant containing pseudotypes, with a final concentration of 4 μg/mL of Polybrene, and incubated at 37°C for 72 h. The cells were then detached from the plate with trypsin, fixed with 4% paraformaldehyde in PBS, and analyzed on a FACSCanto II instrument for GFP expression.

### Neutralization assay.

Twofold serial dilutions of tamarin sera, starting at 1:20 in complete medium, were incubated with a pseudotyped virus input of 1 × 10^5^ RLU per well for 1 h at 37°C. The serum-pseudotyped virus mix was added to Huh7.5 cells seeded the day before at 15,000 cells per well into a 96-well plate with 4 μg/mL of Polybrene. The plates were spin inoculated at 1,240 RCF for 30 min at 20°C and incubated at 37°C for approximately 72 h. Infection was detected as described above, and the RLU were used to calculate the percentage of neutralization by normalizing the results to the values for the pseudotyped virus only (0% neutralization) and cells only (100% neutralization). Dose-response curves were calculated using the percent neutralization against the logarithm-transformed dose fitted to a nonlinear, 4-parameter logistic curve model in GraphPad Prism v.9.1.
